# Impact of meteorological factors on the incidence of hand-foot-mouth disease in Yangzhou from 2017 to 2022: a time series study

**DOI:** 10.3389/fpubh.2023.1278516

**Published:** 2023-10-10

**Authors:** Zaijin Guo, Yin Wang, Yunshui Li, Luojing Zhou

**Affiliations:** ^1^Clinical Medical College, Yangzhou University, Yangzhou, China; ^2^Northern Jiangsu People’s Hospital, Yangzhou, China; ^3^Department of Acute Infectious Disease Control and Prevention, Yangzhou Centre for Disease Control and Prevention, Yangzhou, China

**Keywords:** hand, foot and mouth disease, meteorological factors, generalized additive model, distributed lag nonlinear model, China

## Abstract

**Background:**

Hand, foot, and mouth disease (HFMD) is a significant public health issue in China, and numerous studies have indicated a close association between HFMD incidence and meteorological factors. This study aims to investigate the relationship between meteorological factors and HFMD in Yangzhou City, Jiangsu Province, China.

**Methods:**

HFMD case reports and meteorological data from Yangzhou City between 2017 and 2022 were extracted from the National Notifiable Infectious Disease Surveillance System and the Meteorological Data Sharing Service System, respectively. A generalized additive model (GAM) was employed to assess the exposure-response relationship between meteorological factors and HFMD. Subsequently, a distributed lag nonlinear model (DLNM) was used to explore the exposure-lag-effect of meteorological factors on HFMD.

**Results:**

HFMD in Yangzhou City exhibits obvious seasonality and periodicity. There is an inverted “U” shaped relationship between average temperature and the risk of HFMD, with the maximum lag effect observed at a temperature of 25°C with lag 0 day (RR = 2.07, 95% CI: 1.74–2.47). As the duration of sunshine and relative humidity increase, the risk of HFMD continuously rises, with the maximum lag effect observed at a sunshine duration of 12.4 h with a lag of 14 days (RR = 2.10, 95% CI: 1.17–3.77), and a relative humidity of 28% with a lag of 14 days (RR = 1.21, 95% CI: 1.01–1.64). There is a “U” shaped relationship between average atmospheric pressure and the risk of HFMD, with the maximum effect observed at an atmospheric pressure of 989 hPa with no lag (RR = 1.45, 95% CI: 1.25–1.69). As precipitation increases, the risk of HFMD decreases, with the maximum effect observed at a precipitation of 151 mm with a lag of 14 days (RR = 1.45, 95% CI: 1.19–2.53).

**Conclusion:**

Meteorological factors including average temperature, average atmospheric pressure, relative humidity, precipitation, and sunshine duration significantly influenced the risk of HFMD in Yangzhou City. Effective prevention measures for HFMD should be implemented, taking into account the local climate conditions.

## Introduction

Hand, foot, and mouth disease (HFMD) is an acute infectious disease caused by enteroviruses, primarily Coxsackievirus A16 (CV A16) and Enterovirus 71 (EV71). HFMD is characterized by the development of characteristic lesions on the hands, feet, and mouth. The transmission routes of HFMD mainly include contact transmission, respiratory transmission, and fecal-oral transmission. This disease predominantly affects children under the age of 5 (1, 2). The majority of patients with HFMD present with symptoms such as fever, as well as vesicular or popular eruptions on the hands, feet, and oral mucosa. The prognosis is generally favorable, with self-resolution occurring within approximately 1 week. However, a small proportion of patients may develop severe complications, including aseptic meningitis, encephalitis, pulmonary edema, and other severe manifestations. In rare cases, HFMD can progress to a critical condition and even result in death (3, 4). In recent years, the Asia-Pacific region has experienced frequent outbreaks of HFMD, posing a significant threat to the lives and health of children and adolescents in affected countries. These outbreaks have also imposed a substantial disease burden on the social and economic aspects of these nations (5–7).

Meteorological factors play a pivotal role in the transmission and epidemiology of infectious diseases, and they have been identified as significant risk factors contributing to the spread of HFMD (8–10), being a seasonal infectious disease, exhibits distinct patterns during specific periods in various regions and countries (11). Notably, several Asian countries, including Japan, Singapore, and mainland China, have observed a seasonal occurrence pattern of HFMD (12). Researchers from diverse countries and regions have conducted investigations on the influence of climate on HFMD, encompassing factors such as temperature, sunshine duration, relative humidity, wind speed, and precipitation. However, the findings from these studies exhibit some inconsistencies (13, 14). Analyzing the impact of meteorological factors on the incidence of HFMD can yield valuable insights for future prevention and control endeavors. The conclusions derived from such analyses can effectively guide the development of appropriate intervention measures.

It has been established that meteorological factors possess the capacity to exert a significant influence on the transmission and dissemination of HFMD, primarily by modulating the behavioral patterns of the pathogens or the hosts involved (15). The distributed lag nonlinear model (DLNM) can be employed to investigate the relationship between meteorological factors and HFMD. Meteorological factors such as temperature, humidity, and rainfall may exhibit certain associations with the incidence rate of HFMD. By utilizing the DLNM model, meteorological factors can be treated as independent variables, while the incidence rate of HFMD serves as the dependent variable. This model incorporates lag terms and nonlinear functions to capture the delayed effects of HFMD incidence and the nonlinear relationship with meteorological factors. The advantages of DLNM include its ability to address non-linear and delayed associations, such as exposure-lag-response, through cross-basis functions. Additionally, DLNM can automatically handle various regression function models such as linear models (LM), generalized linear models (GLM), and generalized additive models (GAM). A study conducted in Beijing utilizing a case-crossover design and DLNM revealed a non-linear relationship between temperature and hand, foot, and mouth disease (HFMD), indicating that the risk of HFMD increases with rising temperatures, with the highest risk observed at 25°C–27°C (16). Another study conducted in Sichuan Province using DLNM identified the interactive effects of meteorological factors and air pollutants on HFMD, highlighting that the combined presence of SO_2_ and high temperature and humidity exerted the strongest impact on HFMD (17). Laboratory and epidemiological research has also demonstrated the crucial role of humidity in the transmission of HFMD, with relative humidity accounting for over 84% of the impact on pediatric HFMD, and each 1% increase in relative humidity associated with a 0.34% increase in pediatric HFMD (18, 19). A study conducted in Vietnam demonstrated that an increase in average rainfall is associated with an increased risk of HFMD, an increase in 1 unit of rainfall was associated with a 0.5% increase of HFMD rate on the lag 1 and 6 days (20). A previous study indicated that diurnal temperature range altered the relationship between temperature and pediatric HFMD, with a higher diurnal temperature range associated with a greater risk of HFMD (21), another study found that climate indicators specific to certain cities, including temperature, sunshine duration, and atmospheric pressure, modified the relationship between relative humidity and HFMD, with an overall pooled humidity-HFMD relationship displaying an approximate U-shaped curve with substantial spatial heterogeneity (*I*^2^ = 77.8%), and a reference relative humidity of 70% associated with an RR value of 0.83 (22).

Since being classified as a Class C notifiable infectious disease in China in 2008 (23), HFMD has consistently ranked high in terms of reported cases and fatalities. The generalized additive model (GAM), a flexible and effective approach for parametric, non-parametric, and semi-parametric regression analysis, has been widely employed in time series studies and can be used to identify the relationships between meteorological factors and infectious diseases such as bacterial dysentery, mumps, and hemorrhagic fever with renal syndrome (24, 25).

In recent years, the incidence rate of HFMD in Yangzhou City has remained high, making it the leading notifiable infectious disease in the region (26). This study aims to describe the epidemiological trends of HFMD in Yangzhou City from 2017 to 2022. The GAM will be employed to explore the exposure-response relationship between meteorological factors and HFMD. Additionally, DLNM will be used to assess the exposure-lag-response effects of meteorological factors on HFMD.

## Data and methods

### Study area

**Figure 1:**
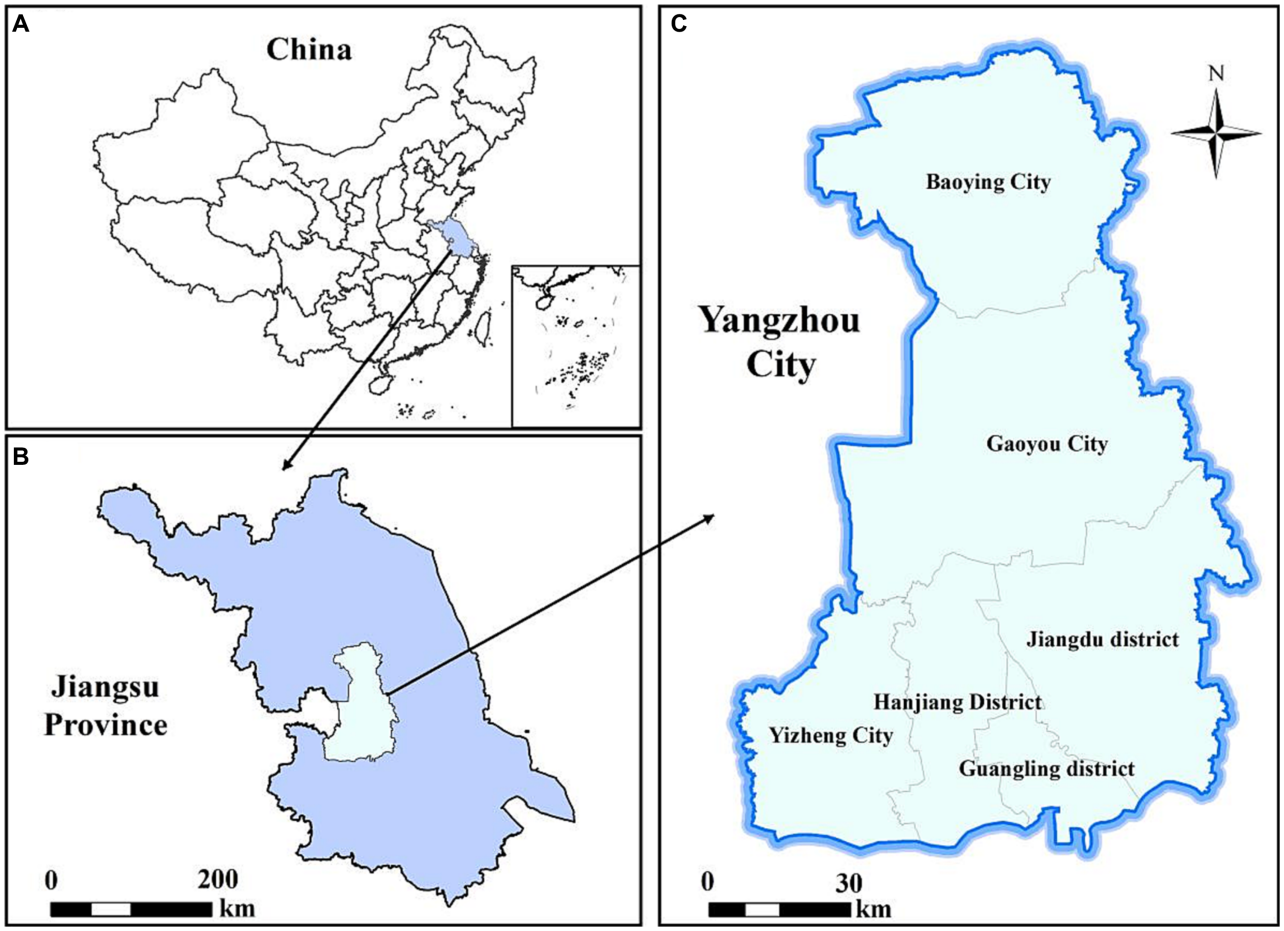
Geographical location of Yangzhou City, China. **(A)** China; **(B)** Jiangsu Province; **(C)** Yangzhou City.

Yangzhou, situated at the heart of Jiangsu Province, assumes a pivotal role in the advancement of the Yangtze River Economic Belt. Geographically, this city spans an expansive area of 6591.21 square kilometers and is demarcated into three districts, one county, and two county-level cities, as visually depicted in Figure 1. The climatic conditions in Yangzhou exhibit distinctive seasonal variations, characterized by copious precipitation, ample sunshine, and discernible shifts in wind patterns corresponding to the changing seasons. Notably, no reports have been documented regarding the influence of meteorological factors on HFMD and its predictive capabilities within the confines of Yangzhou. Consequently, it becomes imperative to comprehend the precise impact of meteorological factors on HFMD within the context of Yangzhou.

### HFMD and meteorological data

The daily reported data on HFMD cases in Yangzhou City from 2017 to 2022 were obtained from the National Infectious Disease Information Monitoring and Reporting Management System. HFMD is classified as a Class C notifiable infectious disease, and all cases diagnosed by qualified doctors in hospitals at all levels nationwide are required to be reported through this system within 24 h. According to the HFMD diagnostic guidelines issued by the Chinese Ministry of Health, all HFMD cases are diagnosed based on clinical symptoms and laboratory test results. The information for each reported HFMD case includes a case number, gender, age, population category, date of onset, and residential address. The meteorological data used in this study was obtained from the China Meteorological Data Sharing Service System.^1^

### Statistical methods

#### Spearman correlation analysis

Spearman’s rank correlation analysis is a non-parametric statistical method used to assess the correlation between two variables. The formula for Spearman’s rank correlation coefficient, denoted as *r*_s_, is as follows:


$$r_{s}=1-\frac{6\sum_{i=1}^{n}d_{i}^{2}}{n\left(n^{2}-1\right)}$$

where *r*_s_ represents the Spearman’s rank correlation coefficient, *n* represents the sample size, and di represents the difference in ranks between the two sets of data. The coefficient ranges from −1 to 1, where −1 indicates a perfect negative correlation, 0 indicates no correlation, and 1 indicates a perfect positive correlation. In this study, Spearman’s analysis was employed to evaluate the correlation between meteorological factors and the incidence rate of HFMD, in order to determine which meteorological factors may have an impact on the disease incidence rate.

#### Generalized additive model

Given the non-linear relationship between HFMD and meteorological factors, as well as the monthly periodicity of HFMD incidence, we applied a generalized additive model (GAM) to estimate the impact of meteorological parameters on monthly HFMD cases (27). GAM is helpful in determining the exposure-response relationships of various types of data, particularly when exploring non-parametric relationships (28). The dependent variable in this study is the cases of HFMD in Yangzhou city, which is a small probability event relative to the whole Yangzhou city population, and can be approximated as its obeying Poisson distribution, so the LOG function is chosen as the link function. In addition, as the spline function has strong ability to apply data and function transformation, and has certain overall smoothness, it is an ideal tool for function approximation, so the function corresponding to each independent variable in this study is set as the spline function, and the model is as follows:


$$\log\left[E\left(Y_{t}\right)\right]=\alpha +s\left(tl,df\right)+s\left(ts,df\right)+\overset{k}{\underset{i=1}{\sum }}s\left(X_{i},df\right)$$

*Y*_t_ is the number of HFMD cases in month *t*; *E*(*Y*_t_) is the expected value of HFMD cases in month *t*; *α* is the constant term of the model; *s*() denotes the spline function; *tl* is used to control for long-term trends; *ts* is used to control for seasonal trends *X_i_* denotes the independent variable (contemporaneous meteorological factors); *df* denotes the degrees of freedom on the spline function of the independent variable, obtained from generalized cross-validation.

#### Distributed lag non-linear model

It is used to quantitatively assess the “exposure-lag-effect” relationships between variables. The DLNM model builds upon the framework of traditional models by utilizing cross-basis functions, allowing for the simultaneous modeling of non-linear and lagged effects in exposure-response relationships. It has been widely employed in studying the associations between environmental exposures and infectious diseases (29). The model is as follows:


$$\begin{array}{c} Y_{t}\sim \mathrm{Poisson}\left(u\right)=\alpha^{*}cb\left(M,df,lag,df\right)+\sum ns\left(X_{i},df\right)\\ \;\;\;\;\;\;\;\;\;\;\;\;\;\;\;\;\;\;\;\;\;\;\;\;\;\;\;\;\;\;\;+ns\left(\mathrm{Time,}df\right)+\beta {\mathrm{DOW}}_{t} \end{array}$$

In the model, *t* represents the observation date, *Y*_t_ represents the number of HFMD cases on day *t*, *α* represents the intercept, *cb* represents the cross-basis functions used to assess the non-linear relationship and lagged effects between meteorological factors and HFMD cases, ns represents natural cubic spline functions, *M* represents the study variables (various meteorological factors), *X_i_* refers to other factors except *M* in the model to control the confounding effect. Time represents seasonal and long-term trends, and DOW refers to the day of the week effect. Based on the incubation period of HFMD and existing research (30, 31), set maximum lag days lag to 14.

## Results

### Characterization of research data

**Figure 2:**
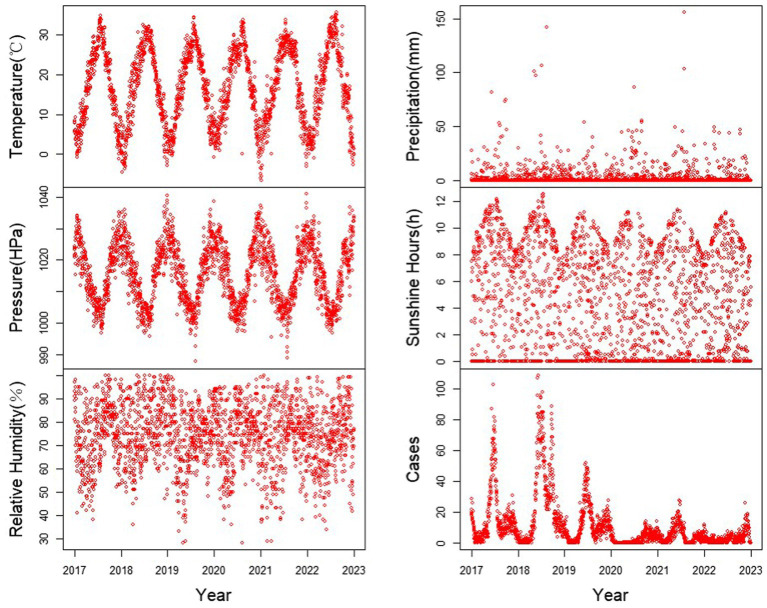
Time series of meteorological factors and number of HFMD cases in Yangzhou City, 2017–2022.

**Figure 3:**
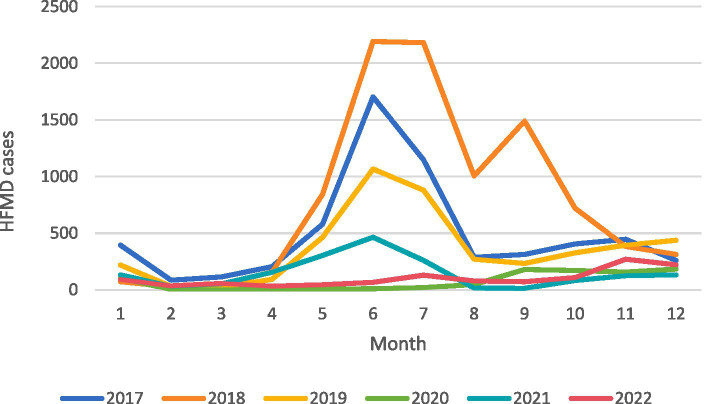
Number of cases of hand, foot and mouth disease in Yangzhou City, 2017–2022.

From January 1, 2017, to December 31, 2022, a total of 23,652 cases of HFMD were reported in Yangzhou City. Among these cases, there were 13,858 male and 9,794 female patients. The annual reported cases were 5,935, 9,431, 4,437, 884, 1,765, and 1,200, exhibiting a pattern of “high-low” years. During the same period, the daily average temperature, atmospheric pressure, relative humidity, precipitation, and sunshine duration in Yangzhou City were 16.81°C (range: −6.5°C to −35.7°C), 1014.94 KPa (range: 999–1041.1 hPa), 74.19% (range: 28–100%), 2.83 mm (range: 0.1–156.2 mm), and 4.7 h (range: 0–12.6 h), respectively. Table 1 and Figure 2 summarize the basic information of HFMD cases and meteorological data. Figure 3 depicts the monthly distribution of HFMD cases in Yangzhou City.

**Table 1:** Descriptive study of HFMD and meteorological factors in Yangzhou City, 2017–2022.

Variables	Mean	Min	25th	50th	75th	Max
Number of cases/day	15	0	2	7	15	109
Mean temperature (°C)	16.81	−6.5	8.7	17.0	25.1	35.7
Atmospheric pressure (hPa)	1014.94	989	1007.1	1015.8	1023.0	1041.1
Relative humidity (%)	74.19	28	65	75	84	100
Precipitation (mm)	2.83	0	0	0	0.5	156.2
Sunshine hours (h)	4.70	0	0.2	4.85	8.3	12.6

### Analysis of the correlation between hand-foot-mouth disease and meteorological factors in Yangzhou

The Spearman correlation analysis matrix is presented in Table 2. In this study, temperature, relative humidity, precipitation, and sunshine duration showed positive correlations with the number of HFMD cases, while atmospheric pressure exhibited a negative correlation. Among these variables, temperature, atmospheric pressure, and precipitation were significantly correlated with HFMD cases (*p* < 0.05), while relative humidity and sunshine duration showed no statistically significant association (*p* > 0.05). Temperature had the highest correlation coefficient with HFMD cases (*r* = 0.37), followed by atmospheric pressure (*r* = −0.36).

**Table 2:** Correlation analysis of meteorological factors, hand, foot and mouth disease cases in Yangzhou City.

Variables	Case	Temperature	Pressure	Relative humidity	Precipitation	Sunshine hours
Case	1					
Temperature	0.37^*^	1				
Pressure	0.37^*^	−0.89^*^	1			
Relative humidity	0.027	0.072^*^	−0.061^*^	1		
Precipitation	0.043^*^	−0.28^*^	−0.20^*^	0.35^*^	1	
Sunshine hours	0.032	0.14^*^	−0.03	−0.61	−0.28^*^	1

^*^p < 0.05.

### Exposure-response relationship between meteorological factors and hand, foot and mouth disease

**Figure 4:**
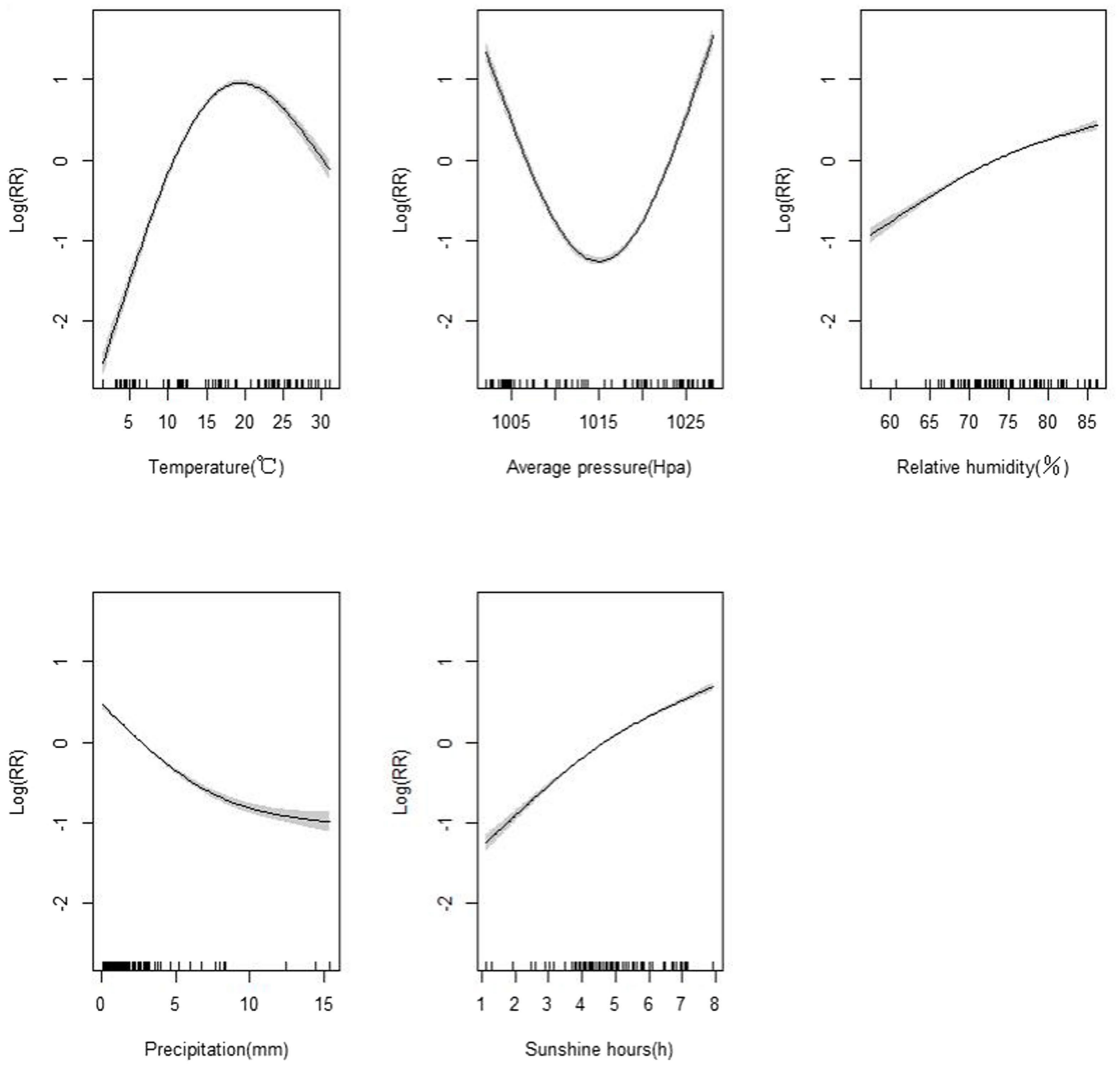
Exposure-response curve of meteorological factors and hand, foot and mouth disease.

The results of the generalized additive model are shown in Figure 4. It can be observed that temperature exhibits a “inverted U-shaped” relationship with the incidence rate of HFMD. As temperature increases, the risk of HFMD initially rises, reaching its peak around 17°C, and then decreases. Atmospheric pressure shows a “U-shaped” relationship with the incidence rate of HFMD. With increasing atmospheric pressure, the risk of HFMD initially decreases, reaching its lowest point around 1,016 KPa, and then increases again. Relative humidity and sunshine duration have similar response on the risk of HFMD, showing a predominantly linear relationship. As relative humidity and sunshine duration increase, the risk of HFMD also increases. On the other hand, the risk of HFMD decreases with increasing precipitation, which is contrary to the effects of relative humidity and sunshine duration.

### Distributed lag nonlinear model

**Figure 5:**
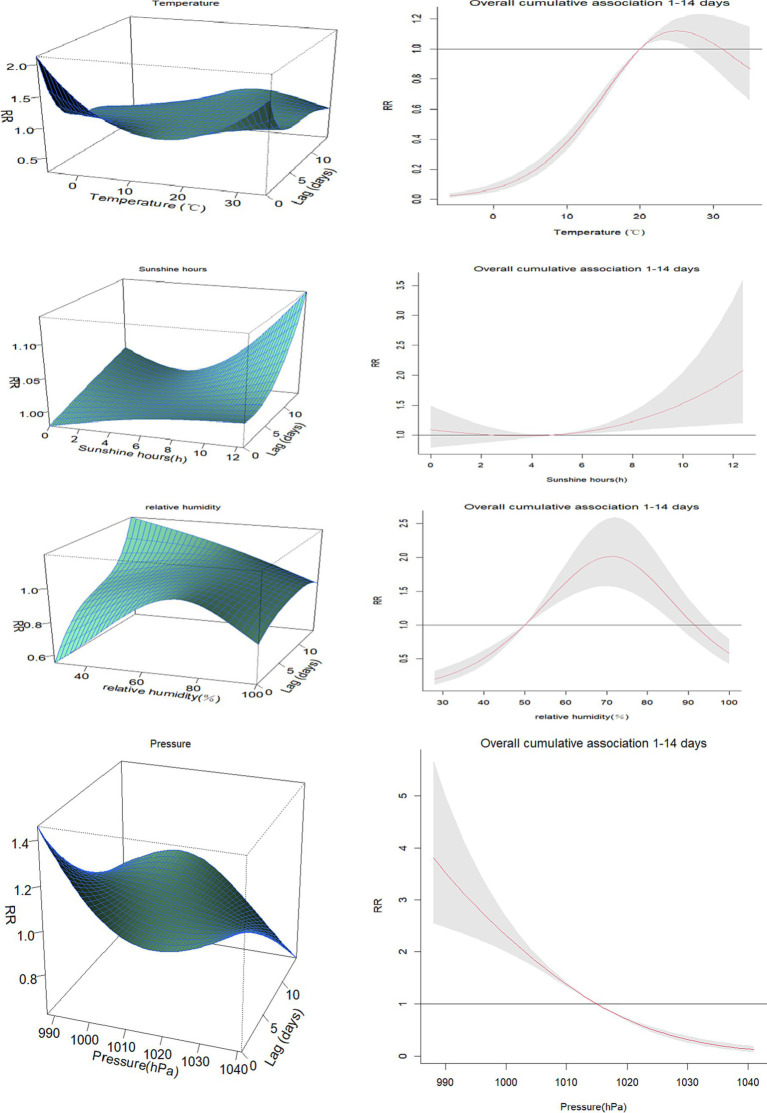

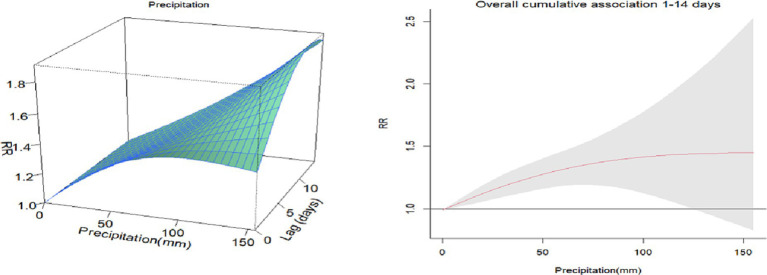
3D plots and cumulative lag effect plots of the impacts of meteorological factors on the risk of HFMD.

We presented the exposure-lag-effect relationships between various meteorological factors and HFMD using a three-dimensional plot. Additionally, we depicted the cumulative effects at the maximum lag of 14 days (Figure 5).

The impact of temperature on HFMD at the maximum lag of 0 days showed that the highest relative risk (RR) value was observed at a temperature of −6°C (RR = 2.07, 95% CI: 1.74–2.47). Using the median temperature as the reference, the cumulative effect of temperature on HFMD approximately followed an inverted U-shaped curve. The maximum cumulative effect was observed at around 25°C (RR = 1.12, 95% CI: 1.05–1.23).

The impact of sunshine duration on HFMD at the maximum lag of 14 days was observed to be highest at 12.4 h (RR = 2.10, 95% CI: 1.17–3.77). Using the median sunshine duration as the reference, the cumulative effect of sunshine duration on HFMD gradually increased. The maximum cumulative effect was observed at 12.6 h of sunshine duration (RR = 2.08, 95% CI: 1.20–3.60).

The impact of relative humidity on HFMD at the maximum lag of 14 days was found to be highest at 28% (RR = 1.21, 95% CI: 1.01–1.64). Using the median relative humidity as the reference, the cumulative effect of relative humidity on HFMD exhibited an inverted U-shaped pattern. As relative humidity increased, the RR value initially increased and then decreased. The peak cumulative effect was observed at a relative humidity of 70% (RR = 2.01, 95% CI: 1.57–2.59).

The impact of atmospheric pressure on HFMD at the maximum lag of 0 days was found to be highest at 989 hPa (RR = 1.45, 95% CI: 1.25–1.69). Using the median atmospheric pressure as the reference, the cumulative effect of atmospheric pressure on HFMD gradually decreased. The maximum cumulative effect was observed at an atmospheric pressure of 990 hPa (RR = 3.79, 95% CI: 2.54–5.66).

The impact of precipitation on HFMD at the maximum lag of 14 days was found to be highest at 151 mm (RR = 1.90, 95% CI: 1.30–3.88). Using the median precipitation as the reference, the cumulative effect of precipitation on HFMD increased initially and then leveled off as precipitation … increased. The maximum cumulative effect was observed at a precipitation of 156 mm (RR = 1.45, 95% CI: 1.19–2.53).

## Discussion

Since its first reported case in New Zealand in 1957, HFMD has rapidly spread to most countries and regions worldwide. In China, HFMD has become a significant public health issue since 2008. The Chinese government has implemented a series of measures to address this problem, including strengthening surveillance and reporting systems, enhancing vaccine research and promotion, and improving public education and health campaigns. Despite some achievements, HFMD remains an important challenge in China, requiring continuous attention and efforts to combat it (32). The impact of meteorological factors on human health has received extensive attention and is closely associated with the occurrence and transmission of various infectious diseases (27, 33, 34). The aim of this study is to investigate the relationship between HFMD cases and meteorological factors, examining the impact of meteorological factors on HFMD from two temporal dimensions: monthly data and daily data.

We conducted an observational analysis of HFMD cases and meteorological data in Yangzhou City from 2017 to 2022. The study revealed that HFMD in Yangzhou City exhibits clear seasonality and periodicity, with a bimodal distribution: the onset of cases begins in May, with the first peak occurring in June and a smaller peak in August. Overall, HFMD has a higher incidence during the summer and autumn seasons, while it decreases during the winter and spring seasons. In terms of epidemic years, HFMD showed a high incidence from 2017 to 2019, followed by a decline likely influenced by the COVID-19 pandemic. These findings are consistent with studies conducted in other Provinces of China (35, 36).

The impact of meteorological factors on HFMD is believed to be influenced by the intricate interplay among the pathogen, environmental factors, and the host population (22). Our research findings have revealed a non-linear relationship, characterized by an “inverted U-shaped” curve, between the average temperature and the incidence of HFMD. This implies that the risk of HFMD tends to be lower at extremely low and high temperatures, while it is higher within the temperature range that is more conducive to disease transmission. The DLNM also revealed that the cumulative effect is highest at 25°C during the 14 days lag period. It is important to note that our research outcomes may diverge from studies conducted in other regions of China, where the relationship between average temperature and HFMD may exhibit an “M-shaped” pattern (37, 38). Moreover, we have also observed a positive correlation between relative humidity and the occurrence of HFMD. On one hand, in conditions of high relative humidity, the pathogens associated with HFMD may thrive, endure for longer durations, and exhibit heightened infectivity. On the other hand, elevated relative humidity can impede sweating and disrupt the metabolic processes in children. This finding aligns with research conducted in other regions of China.

Our study has also unveiled a U-shaped correlation between average atmospheric pressure and HFMD, albeit without the ability to definitively establish a causal relationship between the two. In terms of sunshine duration, our results show that there is a positive relationship between hand-foot-mouth disease and sunshine duration. As the duration of sunshine increases, the risk of HFMD escalates accordingly. It is worth noting that this conclusion contradicts the findings of other studies (39). Conversely, as precipitation levels rise, the risk of HFMD diminishes. This finding aligns with research conducted in other regions of China (40, 41). The reduced risk of HFMD with increased precipitation can plausibly be attributed to the unfavorable conditions for the survival of enteroviruses in high rainfall. Moreover, during periods of heavy precipitation, children may exhibit reduced inclination towards outdoor activities, thereby minimizing their exposure to the virus. Consequently, it is imperative to remain vigilant during periods characterized by high levels of sunshine duration and precipitation. The varying impact of meteorological factors on HFMD across different regions can be attributed to factors such as climate variations, disparities in viral strains, and divergent population behaviors among these regions (42).

This is the first study to explore the impact of meteorological conditions in Yangzhou City on the association with HFMD, expanding our understanding of the influence of meteorological factors on the risk of HFMD. Our research findings have practical implications in two aspects. Firstly, in the formulation of public health policies, our study results indicate that meteorological conditions affect the incidence rate of HFMD in Yangzhou City. For example, high rainfall and prolonged sunshine hours are associated with an increased incidence rate of HFMD, suggesting the need for different policies during the rainy season and dry season. Secondly, in the development of individual-level intervention measures, our research can serve as a reference. Children should develop healthy hygiene habits, such as washing hands before meals and after using the restroom. During HFMD outbreaks, parents or guardians should pay attention to reducing children’s outdoor activities. Additionally, it is necessary to check weather forecasts and air quality before going out.

However, this study has certain limitations. Firstly, time series analysis is an ecological study and may be susceptible to ecological fallacy. This study only focuses on the overall population and does not stratify by gender or pathogen. Secondly, the epidemic process of HFMD is influenced by both natural and social factors (43). Despite the paramount importance of meteorological factors in the transmission dynamics of HFMD, it is imperative to acknowledge that social behavior, economic factors, population mobility, and air quality may also exert significant influences on the occurrence and dissemination of the disease. Regrettably, our study did not encompass an examination of these multifaceted factors, thereby limiting the comprehensive understanding of the complex interplay between various determinants and the epidemiology of HFMD.

## Conclusion

Meteorological factors such as average temperature, average atmospheric pressure, relative humidity, precipitation, and sunshine duration have a significant impact on the risk of HFMD in Yangzhou City. The relationship between average temperature and HFMD risk follows an inverted U-shaped pattern, while the relationship between average atmospheric pressure and HFMD risk exhibits a U-shaped pattern. The risk of HFMD continuously increases with increasing relative humidity and sunshine duration, while it gradually decreases with increasing precipitation, showing a negative correlation. Our study fills a research gap regarding the impact of meteorological factors on HFMD in Yangzhou City. These findings can provide scientific evidence for relevant authorities to implement preventive measures and offer practical recommendations for establishing an early warning and prevention system for infectious diseases.

## Data availability statement

The raw data supporting the conclusions of this article will be made available by the authors, without undue reservation.

## Author contributions

ZG: Writing – original draft, Writing – review & editing. YW: Data curation, Methodology, Project administration, Writing – original draft. YL: Conceptualization, Investigation, Software, Writing – review & editing. LZ: Funding acquisition, Validation, Writing – original draft, Writing – review & editing.

## Footnotes

^1^http://data.cma.gov.cn/
